# Reconsidering screening thresholds in health assessments for obstructive sleep apnea using operational and safety incident data

**DOI:** 10.1038/s41598-024-61118-y

**Published:** 2024-05-13

**Authors:** Anjum Naweed, Bastien Lechat, Janine Chapman, Robert J. Adams, Sally A. Ferguson, Armand Casolin, Amy C. Reynolds

**Affiliations:** 1 Appleton Institute for Behavioural Science, Central Queensland University, Wayville, SA 5034 Australia; 2 Flinders Health and Medical Research Institute (Sleep Health), Flinders University, Bedford Park, Australia; 3 Respiratory, Sleep and Ventilation Service, Southern Adelaide Local Health Network, SA Health, Adelaide, Australia; 4 Transport for NSW, Macquarie Park, New South Wales Australia

**Keywords:** Sleep apnea, Sleep-disordered breathing, Occupational health, Mandatory testing, Safety management, Risk factors, Respiratory signs and symptoms, Health policy, Occupational health, Body mass index

## Abstract

The rail industry in Australia screens workers for probable obstructive sleep apnea (OSA) due to known safety risks. However, existing criteria to trigger screening only identify a small proportion of workers with OSA. The current study sought to examine the relationship between OSA risk and rail incidents in real-world data from Australian train drivers, and conducted a proof of concept analysis to determine whether more conservative screening criteria are justified. Health assessment (2016–2018) and subsequent rail incident data (2016–2020) were collected from two passenger rail service providers. Predictors included OSA status (confirmed no OSA with a sleep study, controlled OSA, unknown OSA [no recorded sleep assessment data] and confirmed OSA with no indication of treatment); OSA risk according to the current Standard, and OSA risk according to more conservative clinical markers (BMI threshold and cardiometabolic burden). Coded rail safety incidents involving the train driver were included. Data were analysed using zero-inflated negative binomial models to account for over-dispersion with high 0 counts, and rail safety incidents are reported using Incidence Risk Ratios (IRRs). A total of 751 train drivers, typically middle-aged, overweight to obese and mostly men, were included in analyses. There were 43 (5.7%) drivers with confirmed OSA, 62 (8.2%) with controlled OSA, 13 (1.7%) with confirmed no OSA and 633 (84.4%) drivers with unknown OSA. Of the 633 train drivers with unknown OSA status, 21 (3.3%) met ‘at risk’ criteria for OSA according to the Standard, and incidents were 61% greater (IRR: 1.61, 95% Confidence Interval (CI) 1.02–2.56) in the years following their health assessment compared to drivers who did not meet ‘at risk’ criteria. A more conservative OSA risk status using lower BMI threshold and cardiometabolic burden identified an additional 30 ‘at risk’ train drivers who had 46% greater incidents compared to drivers who did not meet risk criteria (IRR (95% CI) 1.46 (1.00–2.13)). Our more conservative OSA risk criteria identified more workers, with greater prospective incidents. These findings suggest that existing validated tools could be considered in future iterations of the Standard in order to more sensitively screen for OSA.

## Introduction

Obstructive sleep apnea (OSA) is a sleep breathing disorder characterised by intermittent narrowing and/or closure of the upper airway during sleep. In the transport sector, OSA is consistently associated with excessive daytime sleepiness, fatigue, and inattention^1–4^. In the rail industry, the cognitive deficits associated with OSA have been linked with significant safety incidents^5–8^.

In Australia, the need for standardised management of rail worker health became evident in 2003 following a passenger train derailment in Waterfall, New South Wales (NSW), that resulted in seven fatalities after the driver collapsed from a cardiac event at the controls^9^. A year later, the National Transport Commission (NTC) published the National Standard for Health Assessment of Rail Safety Workers (the Standard)^10^, requiring periodic assessments of individual worker health. Worker history includes medical conditions, sleepiness (Epworth Sleepiness Score^11^), AUDIT questionnaire of alcohol consumption^12^, K-10 questionnaire for psychological distress^13^, and smoking status. A history of involvement in accidents or near misses may be provided by the employer. Clinical examination within the Standard involves hearing, vision and musculoskeletal capacity testing, cardiovascular examination, and calculation of overall cardiac risk level based on age, sex, smoking status, blood pressure, fasting cholesterol and diabetic status. Specific to OSA, the Standard currently assesses OSA risk according to a combination of self-reported sleepiness, or work performance indicators of excessive sleepiness, and some clinical markers including body mass index (BMI), blood pressure, and diabetes status.

**Figure 1:**
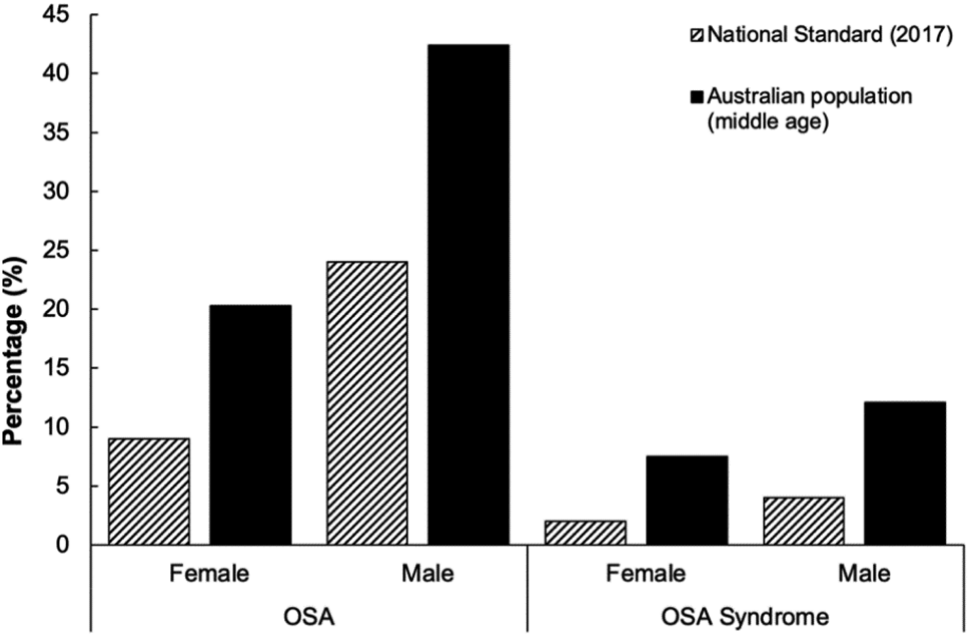
Prevalence rates of clinically significant obstructive sleep apnea (OSA) and OSA syndrome cited in the National Standard for Health Assessment of Rail Safety Workers (2017)^10^ against recent Australian population estimates for middle-aged adults^18^.

Since the Standard was introduced, research has sought to evaluate its application for health screening and clinical assessment^14–16^. A 2015 publication highlighted that OSA within the rail workforce was more prevalent than initially reported, with an increase from 2% in 2009, to 7% in 2012 after revised screening criteria were introduced in the 2012 revision of the Standard^17^. Yet, these rates are still markedly lower than the anticipated prevalence of OSA in this worker population given high rates in the general population^18^. A recent population-level study involving gold standard polysomnography estimated OSA prevalence (based on an apnea hypopnea index (AHI) of >15 or obstructive sleep apnea syndrome, AHI > 5 with excessive sleepiness) as high as 47% in middle-aged males, and 24% in middle-aged females^18^. Thus, the Standard itself still currently references OSA prevalence rates which are >50% lower than recently documented rates (see Fig. 1). This highlights a substantial, hidden burden of undetected OSA in the general population, including in safety critical workers.

In a recent retrospective study of the association between OSA severity and safety incidents occurrence, 44% of rail safety workers with confirmed OSA were found to have at least one incident in the 3 years prior to their health assessment^8^. Undiagnosed and untreated OSA also exposes rail workers to potential major physical and mental health problems. Moreover, the cognitive deficits associated with OSA (e.g., excessive daytime sleepiness, fatigue, and inattention) contribute to ‘operational’ incidents (e.g., failure to stop at a scheduled station, departing earlier than scheduled from the station) which are not safety-related, but still very costly to rail service providers all the same. Without accurately identifying OSA cases in train driver populations, it is not possible to fully elucidate the relationship with safety, nor to appropriately manage the risk.

In order to facilitate evidence-based decisions relating to OSA screening and management, this study used clinical health and rail incident data from train drivers in NSW to answer the overarching research question: what is the relationship between obstructive sleep apnea and prospective rail operational and safety incidents? The aims of the study were to:

1. Compare OSA ‘risk’ status from the Standard with confirmed OSA status to determine whether application of the Standard criteria accurately classifies workers;
2. investigate the association between ‘OSA risk’ as identified in the Standard with real-world rail safety and operational incidents (‘rail incidents’) in Australian train drivers; and to
3. determine whether a more conservative risk threshold for OSA using existing health record data is warranted based on associations with rail incidents following a health assessment.

## Methods

Data were drawn from two passenger rail service providers in NSW, Australia. An *a priori* decision was made to consider health assessment data for all train drivers collected in the timeframe 2016–2018, irrespective of sleep study screening status. This decision was made due to the low prevalence rates of OSA in historic studies of rail safety workers, and to allow for analysis of ‘at risk’ but undiagnosed or unknown OSA status relative to subsequent incident data.

All incident data over the period 2016–2020 were provided for train drivers with a health assessment in this timeframe. When completing their health assessment, employees signed individual informed consent for their health data to be accessed for the purposes of audit or research. Data were deidentified after linkage to protect confidentiality of workers. Research ethics approval was provided by Central Queensland University (approval no. 0000023385), and Flinders University (project no. 5174), and all research was performed in accordance with relevant guidelines and regulations.

### OSA status and risk

Three variables were considered as predictors for analysis.

#### OSA status by current gold standard measurement

OSA status was determined from an overnight polysomnographic sleep study during the health assessment for some participants. Others had existing diagnoses recorded in the health records. Train drivers were coded as either ‘confirmed no OSA’ *(sleep study completed with no diagnosis of OSA)*, ‘controlled OSA’ *(sleep study with diagnosis of OSA plus notes about treatment)*, ‘unknown OSA’ *(train drivers with no recorded sleep study or notes indicative of diagnosis of OSA)*, and ‘confirmed’ OSA *(train drivers with a diagnosis of OSA but no indication of treatment initiation in the 2016-2018 health assessment data).*

#### OSA risk by the current version of the Standard

The second variable was OSA risk according to the Standard (section 18.6, ‘sleep disorders’)^10^. Train drivers were coded as ‘at risk’ according to the Standard if they had demonstrated sleepiness (ESS ≥ 16) *or* body mass index (BMI) >40, or BMI > 35 with comorbid type 2 diabetes or high blood pressure requiring medications for control.

#### OSA risk by conservative clinical markers

The final variable was OSA risk according to clinical markers. For the purpose of this variable, to be ‘at risk’ by BMI category, workers had to meet BMI threshold (≥37.5 kg/m^2^), and/or have a BMI > 35 with an indication of current cardiometabolic burden (e.g., type II diabetes^19^, high blood pressure requiring ≥ 1 medications^20,21^, systolic blood pressure ≥ 135 & diastolic blood pressure ≥ 85 and/or any heart diseases^22^). This more conservative criteria for OSA risk was selected given the associations with OSA in existing literature, and availability of clinical marker data in the cohort to demonstrate proof of concept.

#### Comparison between risk and Gold Standard polysomnography

The classification of OSA risk by the current version of the standard (binary outcome, at risk/not at risk) was compared to OSA diagnosis according to polysomnography (OSA/no OSA) using a confusion matrix as depicted conceptually in Table 1. A true negative is a driver that screened negative according to the Standard criteria, and negative on a sleep study (a “confirmed no OSA”). A false positive is a driver that screened negative using the Standard, but a positive diagnosis using gold polysomnography. Furthermore, we define the misclassification of OSA risk according to the Standard by dividing the number of false positives by the total number of positive cases according to gold-standard polysomnography. For the purpose of this analysis, whether the OSA was controlled/treated is not taken into account, given that a driver would have had a to fulfill criteria in the first place to be subsequently treated. However, we also calculated the false positive probability/misclassification probability for controlled/not-controlled OSA separately. A similar analysis was conducted for OSA risk as defined using more conservative clinical markers instead of the Standard.

**Table 1:** Explanation of confusion matrix used to examine risk against gold standard polysomnography data.

		Gold-standard polysomnography	
		No OSA	OSA
At risk using the Standard	Not at risk	True negative	False positive
	At risk	False negative	True positive

### Operational and safety incidents

**Figure 2:**
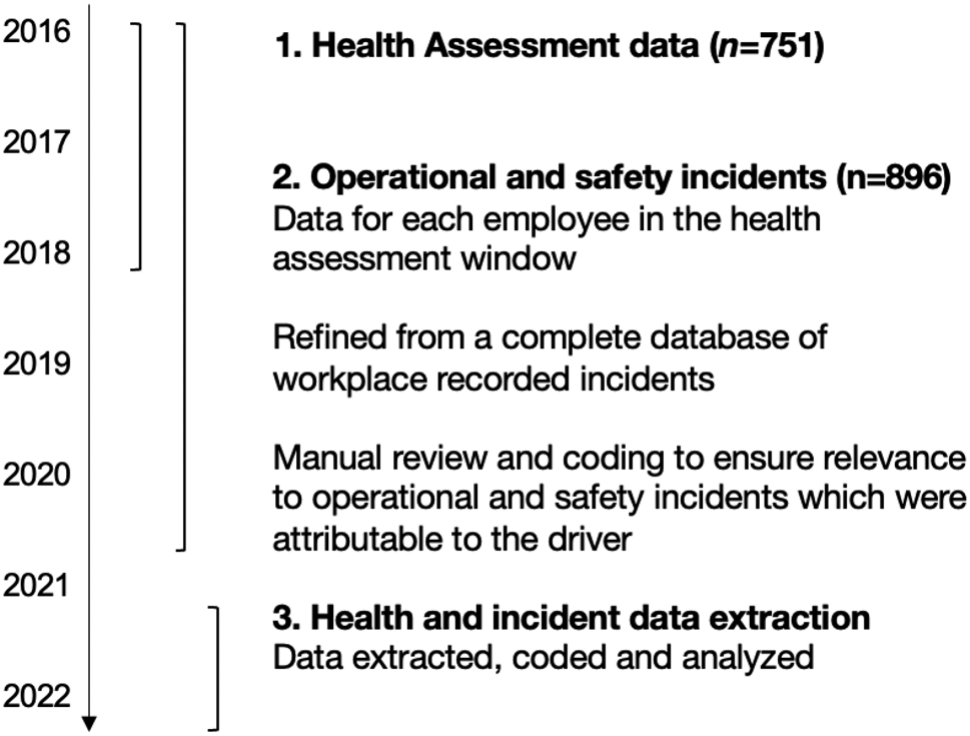
Timeline depicting the health assessment data extraction window in relation to the operational and safety incidents.

The primary outcome in the study was the number of significant operational and safety incidents per worker following a health assessment in the period 2016–2018 (see Fig. 2). Operational and safety incidents were extracted from the Incident Information Management System—a state-wide incident reporting/monitoring system accessed by a wide range of rail professional staff employed by rail service providers in NSW to report incidents. Incidents deemed to be significant enough for inclusion were based on a detailed taxonomy, developed by coding incidents using conventional content analysis^23,24^.

The resulting taxonomy (see Table 2) had 14 overall categories made up of 34 subcategories. Given the range of incident types within the Incident Information Management System, they were also coded as either ‘safety’ (*incidents increasing safety risk and defined primarily by safety impacts*) or ‘operational’ (*incidents impacting scheduling and defined primarily by service impacts*), recognising the dual key performance indicators shaping the rail system, but also the complexity of the relationship between them as they related to health (e.g., Failure to stop at a scheduled station is an operational incident, but can be caused by inattention and microsleeps, both of which are associated with OSA). The process for extraction of incidents including the total number of incidents is summarised in Fig. 2.

**Table 2:** Outline of taxonomy for classifying rail driver-related incidents in the study.

Category of incident	Subcategory	Type of incident
Collision	Train-to-train/railway vehicle collision or near-hit	Safety
	Train-to-infrastructure collision or near-hit	Safety
	Person-train collision or near-hit	Safety
Departure error	Departed station early	Operational
	Departed station late	Operational
	Departed without right of way/flag displayed	Safety
Emergency brake activation	Induced by onboard vigilance system^a^	Safety
	Induced by onboard “deadman” system^b^	Safety
Equipment use	Equipment handling error	Safety
Failure to stop	Signal passed at danger^c^	Safety
	Signal reversion^d^	Safety
	Failure to stop at a scheduled station	Operational
	Overshoot at station of platform/decant facility	Operational
Health management during shift	Relief from shift requested due to sickness	Operational
	Relinquished duty	Operational
	Feeling shaken and unfit to continue shift	Operational
	Toilet/comfort break resulting in delayed departure	Operational
	Medical attention required	Operational
Health-related work-absence	Illness/sickness	Operational
Not at point of duty	Staff not at their point of duty	Operational
	Staff late arrival to train (driver on wrong train or platform)	Operational
Procedural irregularity/breach	Failure to answer radio	Safety
	Incorrect worksite procedure	Safety
	Unplanned/unsafe train move	Safety
Schedule routing	Wrong route taken by driver	Operational
Short stoppage	Platform short stop	Operational
Speed error	Overspeed on mainline/crossover	Safety
	Overspeed in yard/station/depot	Safety
	Intermediate train stop trip^e^	Safety
Train preparation and management	Wrong trainset prepared/boarded	Operational
	Incorrect set-up for distributed power	Operational
	Incorrect log on	Operational
	Train keys forgotten/lost	Operational
Unscheduled stop	Timetable misread	Operational

^a^The vigilance system is a perception–action device requiring manual driver reset following prolonged inaction.

^b^The “deadman” system is a fail-safe mechanism designed to address driver incapacitation or malaise.

^c^Signal passed at danger or “SPAD,” reflects a failure mode where the train has exceeded its limits of movement and encroached into a section of track without authority.

^d^Signal reversion reflects a sudden change of signal and limited opportunity to prevent a SPAD incident.

^e^Mechanism connected to end of station signals designed to stop the train if approach speed is excessive.

### Additional health status and demographic data

Participant age, sex, hypertension status, heart disease status, diabetes status, ESS^11^ and K-10 scores^13^ were extracted from health records. Blood pressure values (systolic and diastolic) were taken from the cardiovascular examination, and qualitatively from relevant notes provided by clinicians during health assessments, including comments on OSA severity, metrics from sleep studies, and OSA treatment status.

### Data analysis

Data were analysed in R Studio^25^, using the *pscl* package. Prospective count of incidents (i.e., operational and safety) were analysed using zero-inflated negative binomial models. This approach is appropriate when data are over-dispersed with high 0 counts, as is the case with safety critical incidents. This approach has been used previously in accident prediction models^26^. All incident data were adjusted for sex and reported as incidence risk ratios (IRRs). The database reflected all driver data available from two rail providers in the target window, and a retrospective power calculation was not conducted^27^.

## Results

### Workers included in the study

A total of 751 train drivers with health assessment data in the period 2016-2018 were included in analyses. Where workers had multiple assessments in the timeframe, the earliest assessment with sufficient data to calculate risk scores for OSA referral according to the Standard was included in analyses (Supplementary Figure 1 provides a summary of the number of assessments conducted per train driver in the health assessment window).

Table 3 shows the demographic and health characteristics of the sample. Included workers were typically middle-aged, with BMI scores placing them in the overweight or obese categories. Workers were predominantly male, typical of train driving cohorts in Australia^28^. Hypertension and diabetes were common in the sample, particularly in workers with OSA (both controlled OSA and confirmed OSA). Sleepiness scores did not differ across OSA categories, and overall scores were well below both the thresholds for identifying sleepiness according to the Standard^10^, and the typical ESS scores reported for middle-aged Australians in the general population^18^.

**Table 3:** Demographic and health characteristics of workers included in analyses.

	Missing	Confirmed no OSA	Controlled OSA	‘Unknown’ OSA	Confirmed OSA
Number of cases (n)		13	62	633	43
Age (years)*	1	48 (11)	48 (10)	50 (11)	44 (11)
Body Mass Index (kg/m^2^)*	0	36 (5)	38 (6)	29 (6)	39 (5)
Sex	0				
*female*		1 (8%)	4 (6%)	26 (4%)	4 (9)
*male*		12 (92%)	58 (93%)	607 (96%)	39 (91%)
Systolic Blood Pressure*	2	134 (13)	135 (14)	133 (15)	137 (19)
Diastolic Blood Pressure*	20	86 (10)	83 (10)	82 (10)	85 (14)
Hypertensive (yes)	24	3 (23%)	29 (50%)	130 (21%)	13 (31%)
Heart disease (yes)	8	0 (0%)	2 (3%)	19 (3%)	2 (5%)
Diabetes (yes)	11	6 (46%)	19 (31%)	62 (10%)	13 (30%)
Epworth sleepiness scale (ESS) score*	**4**	**0.8 (1.1)**	**1.6 (1.9)**	**1.3 (1.7)**	**1.7 (1.8)**
Kessler 10 score*	**4**	**11.5 (4.7)**	**10.6 (1.5)**	**11.1 (16.1)**	**10.5 (1.2)**
Number of prospective incidents	0	1.15 (1.28)	1.60 (1.90)	1.14 (1.40)	1.37 (1.36)
Health assessment year					
*2016*		3 (23%)	12 (19%)	127 (20%)	10 (23%)
*2017*		4 (31%)	22 (36%)	214 (34%)	15 (35%)
*2018*		6 (46%)	28 (45%)	292 (42%)	18 (42%)

Values reflect *n*(%) unless marked with (*), which reflect mean ± standard deviation. OSA categories were determined according to data provided in individual health assessments. Confirmed ‘no OSA’ respondents were workers who had undergone a sleep study and did not meet criteria for OSA. Controlled OSA accounted for workers with a diagnosis of OSA and evidence of treatment from their health assessments. Unknown OSA meant workers had not undergone a diagnostic sleep study, and had no notes related to OSA or OSA treatment. OSA accounted for workers who had a diagnosis of OSA in their health assessment data, but no reference to treatment regime.

### Aim 1: Compare OSA risk status from the Standard with confirmed OSA to determine whether application of the Standard criteria accurately classifies workers

**Figure 3:**
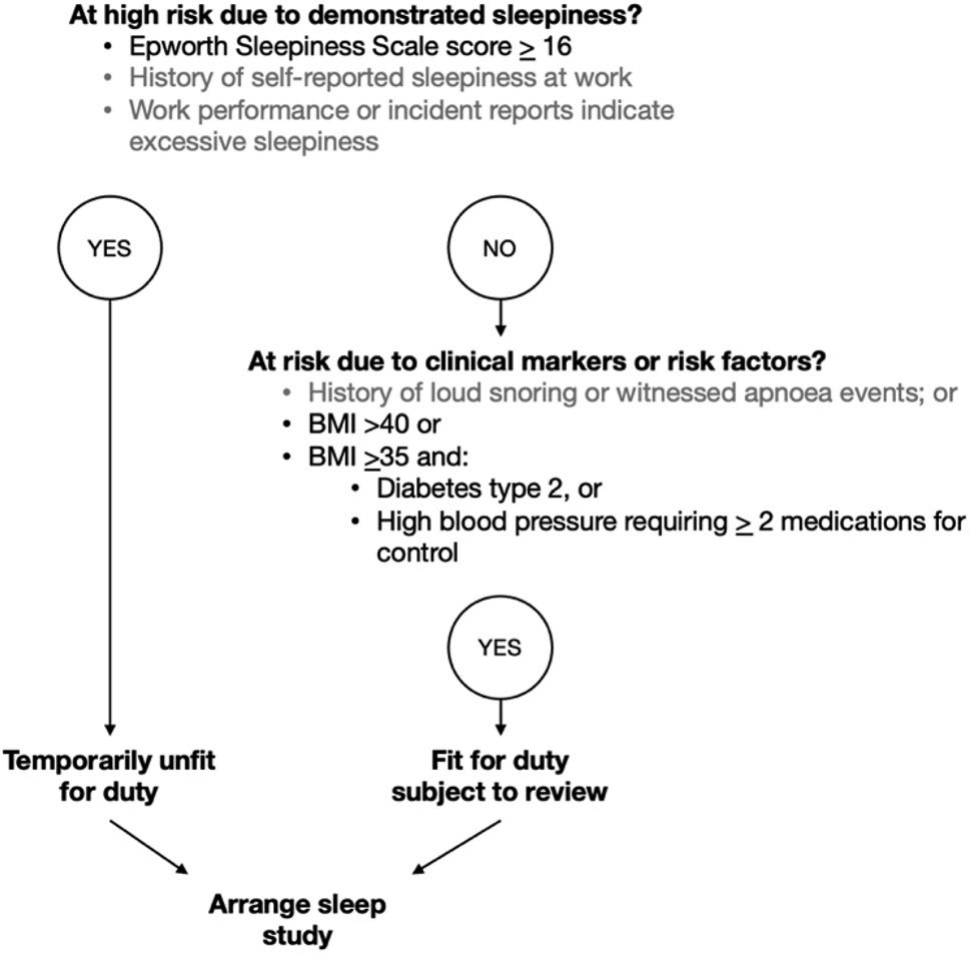
Criteria for referral for a sleep study according to the 2017 National Standard for Health Assessment of Rail Safety Workers^10^. *Note* grey text indicates that subjective details were not consistently recorded or reported in the health assessment data provided. BMI, body mass index.

The Standard relies on both self-reported and objective clinical risk factors to identify workers at risk of OSA. Figure 3 demonstrates available data from health assessments to classify workers as ‘at risk’ of OSA according to the Standard sleep disorder assessment trigger (p. 144 of the Standard^10^). Data were not consistently recorded or available in the current health assessment records to meet all self-reported indicators. As reported in Table 3, mean ESS scores were lower than required for a trigger of temporarily unfit for duty. Review of all 751 train drivers with available health assessment data indicated that no workers met the ≥16 ESS score, even amongst those with a confirmed OSA diagnosis.

Comparison of classification of ‘at risk’ of OSA from the Standard versus polysomnography ‘confirmed’ OSA is provided in Table 4. These findings highlight that the criteria for ‘at risk’ of OSA in the Standard misclassifies 23.2% (10/43) of workers with confirmed OSA (on a sleep study), and 38.7% (24/62) of workers with controlled OSA.

**Table 4:** Confusion matrix highlighting misclassification of OSA risk according to the National Standard for Health Assessment of Rail Safety Workers (2017) against confirmed OSA status derived from health assessment data in 751 workers.

Status from health assessment data	Count of workers who met the ‘at risk’ criteria as given in the Standard	
	No	Yes
Confirmed no OSA	5	8
Controlled OSA	24	38
Confirmed OSA	10	33
Unknown OSA	612	21

*OSA* obstructive sleep apnea.

### Aim 2: Investigate the association between OSA risk as identified in the Standard with real-world rail safety and operational incidents (‘rail incidents’) in Australian train drivers

**Figure 4:**
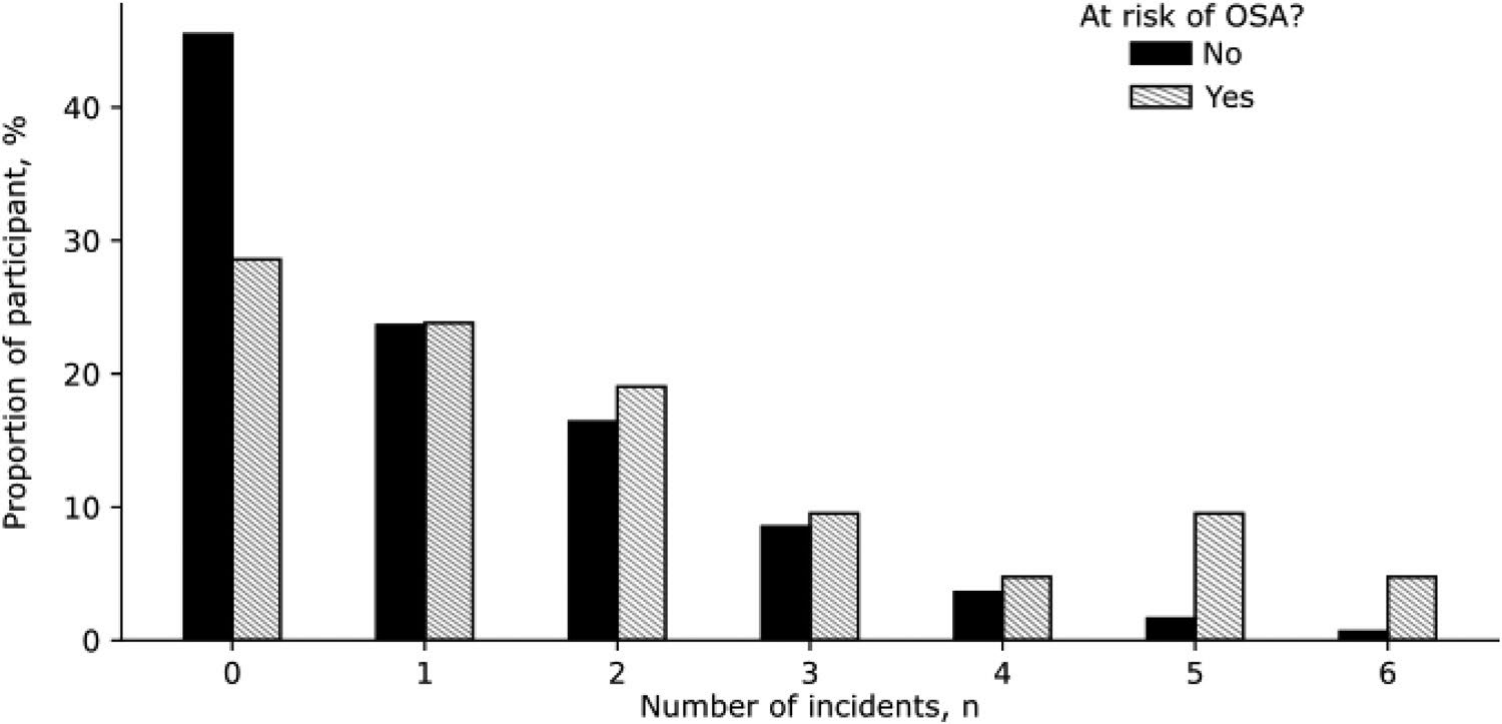
Number of incidents per participant for participant at risk (shaded) and not at risk (black) of OSA according to the current Standard. Note that there is a greater proportion of participants at risk of OSA with more than two events in the 2016–2020 window, and a lower proportion of participants at risk of OSA with no incidents in the timeframe.

To determine whether ‘at risk’ train drivers according to the Standard experienced greater operational and/or safety incidents in the time following the health assessment window, we analysed data from 633 train drivers who had an ‘unknown’ OSA status (workers who had not undergone a sleep study to confirm OSA). A total of 723 of the 896 (80.7%) events were attributable to these 633 drivers. Of these 633 train drivers, 21 (3.3%) met ‘at risk’ criteria for OSA according to the Standard. These ‘at risk’ train drivers had 61% greater incidents in the years following their health assessment compared to drivers who did not meet ‘at risk’ criteria in the unknown OSA group (Fig. 4, Incidence Risk Ratio (IRR): 1.61, 95% Confidence Interval (CI) 1.02–2.56).

### Aim 3: Determine whether a more conservative risk threshold for OSA using existing health record data is warranted based on associations with rail incidents following a health assessment

To determine whether a lower threshold for OSA screening identified significantly greater incidents, we changed the thresholds for the ‘at risk due to clinical markers or risk factors’ trigger criteria. These criteria were drawn directly from available data in the existing health assessment measures. The difference between the trigger criteria in the Standard, and our altered thresholds are demonstrated in Fig. 4.

**Figure 5:**
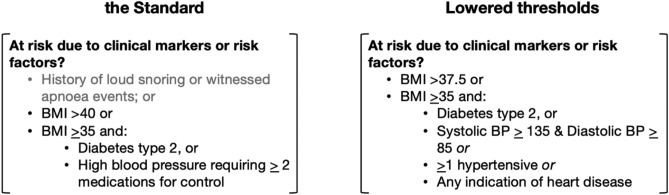
Comparison of existing ‘at risk’ criteria in the National Standard for Health Assessment of Rail Safety Workers^10^ to the lowered thresholds used for re-analysis. *Note* BMI, body mass index, BP, blood pressure.

Application of a lower threshold using existing clinical metrics available in the worker databases, but with more conservative cut points (see Fig. 5), identified an additional 30 ‘at risk’ train drivers, taking the total to 51 (8.1% of the workers with unknown OSA status). Applying these criteria, ‘at risk’ train drivers had 46% greater incidents compared to drivers who did not meet risk criteria (IRR (95% CI) 1.46 (1.00–2.13)).

**Figure 6:**
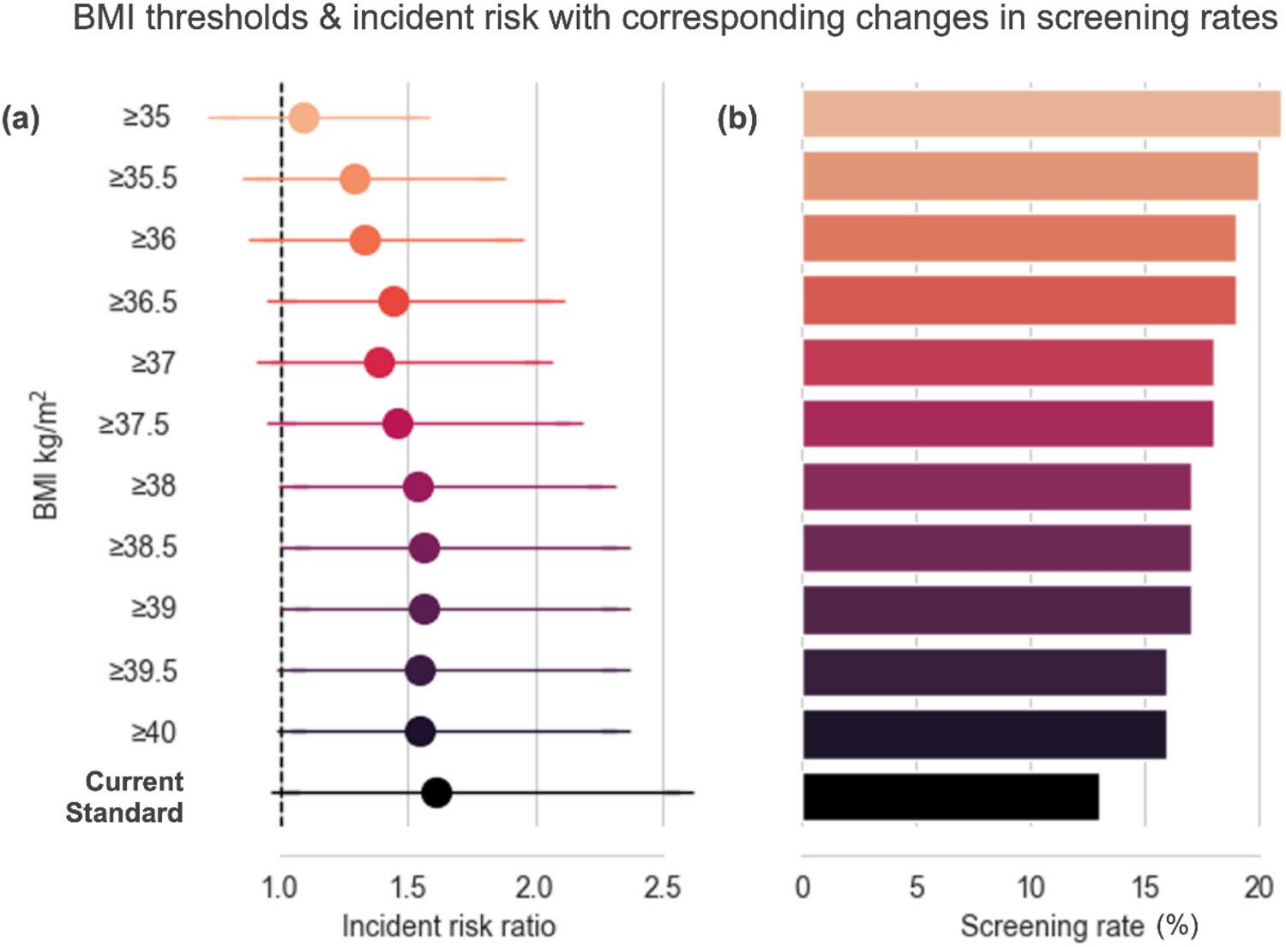
The (**a**) association between different BMI thresholds and risk of (safety and/or operational) incidents, along with (**b**) corresponding changes in screening rates (%) in train drivers. *Note* In Panel A, circles represent the estimand, and lines the 95% confidence intervals. In Panel B, screening rate is presented as a percentage (%) of the unknown OSA category. Data presented are unadjusted. BMI, body mass index.

A final, a posteriori, analysis was conducted to explore the impact of the relationship between OSA risk and operational and/or safety incidents when the BMI cutpoint was varied. Specifically, we set out to determine whether the association with incidents in ‘at risk’ workers remained as BMI cutpoint was progressively reduced. Findings from this analysis are illustrated in Fig. 6, reflecting IRRs and corresponding screening rate as a percentage of workers in the sample if the criteria were amended to the respective BMI levels. These findings are also shown in Table 5, with the indicative ‘greater’ incident rate. Together, these findings demonstrate a dose–response relationship between BMI levels used for screening criteria and prospective incidents likelihood. While 95% CI intervals are relatively large, there is a consistent 40–60% increase in incident likelihood for BMI levels ≥ 36.5 kg/m^2^, and this effect is consistently significant for BMI ≥ 38 kg/m^2^.

**Table 5:** The association between different BMI thresholds and risk of rail incidents with the indicative ‘greater’ incident rates and corresponding changes in screening rates (%) in train drivers.

BMI (kg/m^2^)	IRR (95% CI)	Indicative ‘greater’ incident rate (%)	Screening rate (%)
$\ge$ 35	1.09 (0.77–1.53)	9	21.0
$\ge$ 35.5	1.29 (0.91–1.83)	29	19.8
$\ge$ 36	1.33 (0.93–1.90)	33	19.2
$\ge$ 36.5	1.44 (1.00–2.06)	44	18.6
$\ge$ 37	1.39 (0.96–2.01)	39	18.0
$\ge$ 37.5	1.46 (1.00–2.13)	46	17.8
$\ge$ 38	1.54 (1.05–2.26)	54	17.2
$\ge$ 38.5	1.56 (1.05–2.31)	56	16.9
$\ge$ 39	1.56 (1.06–2.31)	56	16.8
$\ge$ 39.5	1.55 (1.04–2.31)	55	16.4
$\ge$ 40	1.55 (1.04–2.31)	55	16.4
Current Standard	1.61 (1.02–2.56)	61	13.3

Data presented are adjusted for sex. *BMI* body mass index, *IRR* incidence risk ratio, *CI* confidence interval.

## Discussion

This study examined associations between obstructive sleep apnea (OSA) status based on reported OSA diagnosis in health assessments, OSA risk according to clinical risk factors in the existing Standard, and occurrence of rail incidents using real-world data. The parameters currently being used to screen for OSA in the Standard were found to misclassify workers ‘at risk’ of OSA. Specifically, 23.2% of workers with confirmed OSA, and 38.7% of workers with controlled OSA, would not have triggered a sleep study using clinical risk factors similar to the Standard, if they did not have confirmed OSA diagnosis already. We also show greater rail incidents for workers at risk of OSA (but not diagnosed) according to the Standard. Prospective analysis found that lowering the threshold for screening according to clinical risk factors (a combination of lowered BMI and less conservative indications of current clinical markers) identified greater rail incidents. These findings extend recent retrospective analyses which highlight the need to consider more stringent screening of OSA in rail workers^8^, and use more conservative clinical marker thresholds for risk criteria when screening for OSA in train drivers as a proof of concept reflecting the need to review criteria. Future studies should now carefully consider the appropriateness of specific clinical cut points when screening for OSA in larger studies.

The findings of this study extend previous research about the utility and design of the sleep disorder screening component of the current version of the Standard^8^ by highlighting a need to reconsider screening criteria for OSA. While progress has been made in identifying OSA since implementation of the Standard^8,14,17^, OSA prevalence rates in train drivers according to health assessments in these analyses remain markedly lower than prevalence in the general population of similarly aged adults. While one possible interpretation could be that since introduction, workers with OSA are either treated or have left the organisation, this is unlikely as there remain higher rates of risk factors for OSA (including obesity and comorbidities) in this worker demographic than comparably aged adults in Australia^18^. The consequence is high misclassification rates of workers with confirmed OSA and controlled OSA according to health assessment data, and thus the risk of rail incidents attributable to unmanaged OSA is likely still common in train drivers. This was confirmed in the current study by greater incident rates in high-risk workers according to real world data.

Our findings suggest a few key areas for consideration in future revisions of the Standard. One, it is evident from review of the Epworth Sleepiness Scale (ESS) scores collected at worker health assessments that self-reported sleepiness according to the ESS Score is unlikely to add particular value to screening criteria. None of the impacted train drivers in this analysis reported sleepiness scores which would trigger a sleep study, and ESS scores were lower than population averages reported for middle-aged Australians^18^. Workplace health assessments can be perceived with high suspicion in this industry. In some instances, self-reporting that avoids triggering health assessments is the prevailing attitude^16^. Consequently, self-report triggers for health assessment should be applied with caution. Reducing reliance on self-report of sleepiness and increasing use of more conservative physiological status and comorbidity data may facilitate more informed decision making around the need for OSA screening and management. Existing, validated tools such as the STOP-BANG^29^ and others^30,31^ are acceptable, incorporate subjective and objective indicators of OSA, and are easily implemented. Which of these screening criteria better predict OSA risk for rail safety incidents remains to be studied prospectively.

The consequence of revising the current risk thresholds for sleep disorder screening is the unavoidable increase in screening rates and referrals for sleep studies across organisations. This comes with inevitable costs related to diagnosing and managing OSA in worker populations. However, we were able to show that a small adjustment in clinical risk factors (specifically, BMI) reflects a relatively modest increase in screening, whilst also capturing workers at risk for a greater number of incidents. Given that OSA is associated with major incidents, and in some cases fatalities, in the rail industry^32^, the increased costs associated with screening are likely to be offset by the potential to reduce risk of major incidents in train drivers. Management is also likely to improve the negative associations of OSA sickness absenteeism^33^, poorer quality of life^34^, hypertension^20^ and poorer mental health^35^ which are all costly to the individual, the workplace and the health system.

### Strengths, limitations, and future directions

These findings should be considered in light of methodological limitations inherent with interpreting real-world data. A limitation of our analyses is that our altered threshold for OSA ‘at risk’ status was drawn only from available health assessment data. Recording of OSA status, including severity, was reliant on the quality of health assessment notes. For example, the apnea hypopnoea index (AHI) is the indicator used to diagnosis OSA. The AHI was not routinely recorded for all workers with OSA, particularly if the worker was not a new diagnosis of OSA during the study window, and was considered ‘controlled’ OSA. Given that OSA severity is associated with different impacts on road and rail safety, it may be beneficial for authorised health professionals to record the AHI consistently as a metric of OSA severity, to ensure meaningful comparison by severity is feasible in future studies, as this was not possible within the current study. Another limitation is that the provided analyses relied on a specified window of health assessment data (2016-2018), which lead to a modest sample size available for analysis. For the incident analysis, we also assumed that the health assessment used to determine OSA status or risk is an accurate representation, and that this did not change during the subsequent incident period. This meant we were unable to determine whether some workers had sought treatment within the assessed analysis window. Consequently, we recommend that these analyses should be repeated with a larger sample size, including multiple health assessments during the incident period, before definitive conclusions can be drawn about the relationship between OSA risk status and rail safety incidents.

It is also worth considering in future whether emerging technologies could be used to better quantify OSA severity in train drivers, rather than relying on self-report, screeners and/or single night polysomnography (PSG). New metrics of OSA severity, including measures of hypoxia^36,37^, sleep fragmentation^38,39^ and autonomic responses^40,41^ have been shown to better predict cardio-metabolic risk associated with OSA. The implementation of these approaches in this worker population may also be helpful in future for identifying workers at risk of significant incidents, and should be considered where possible in future analyses. Also of relevance, night-to-night variability in OSA severity can be significant, leading to misclassification of disease severity and misdiagnosis in 20–50% of cases when diagnosis is made using single-night PSG^42,43^. Multi-night assessment of OSA severity using low-cost ‘nearable’ sleep measurement devices has been shown to provide more reliable estimates of OSA severity and associated health outcomes compared to traditional PSG^20,44^. Using similar approaches for this worker population may facilitate more detailed analysis of the application of the Standard, and any future amendments or iterations, relative to OSA status in train drivers. This would also facilitate evidence-based continuous improvement of sleep disorder screening recommendations in future, to ensure workers most impacted by OSA are appropriately screened and treated.

The database from which incident data were extracted was very comprehensive. However, we were not able to incorporate detailed information about worker schedules, including shift work schedules, which would be beneficial in future studies given previous findings of OSA syndrome symptoms worsening during daytime sleep for shift workers^45^ which could have implications for driver safety. This limits our ability to further explore the relationship between OSA, shift work schedules and incidents in this sample. We were also not able to account for workers leaving the industry or driving time per week in the current analyses which would be beneficial in future analyses. There was a discordance between our OSA at risk screening criteria and the Standard in 21 participants (3.3%) who should have been screened for OSA. This may be because we were not able to replicate the Standard OSA risk exactly due to missing data in our sample, however, the more likley explanation is that some of these drivers may also have been screened in an earlier clinical assessment, which would have been missed. Further, in the window of interest to this study, incident records were susceptible to change in terminology, detail and classification, including errors and inconsistencies in entry of incident data. This meant that data needed to be coded manually, but is also likely to have resulted in some incidents coded in a fashion which meant relevant operational or safety incident data could not be included. This raises a general need for better and more consistent entry of incident data over time to quantify impacts of health conditions on rail safety incidents more broadly.

## Conclusion

Undiagnosed and unmanaged OSA poses significant health and safety risks for the rail industry. In Australia, a national Standard has been applied to try to manage this risk for some time. However, the utility of this Standard appears to be limited by use of screening questions and risk factor criteria that are likely to only identify the most severe of OSA cases, leaving others undiagnosed and unmanaged. We demonstrated considerable OSA misclassification based on existing Standard criteria for OSA screening compared to confirmed OSA status within the health assessment window. This study also showed a clear relationship between OSA status and subsequent rail incidents in train drivers using the existing Standard. Finally, we demonstrated that lowering the risk threshold according to clinical risk factors for OSA below what is required for the current Standard still identified workers with greater incidents. These results are the first of their kind for rail and suggest a need to reconsider the risk factors and screening approaches for OSA in future iterations of the Standard.

## Data Availability

All data generated or analysed during this study are included in this published article [and its supplementary information files]. Due to data privacy and confidentiality requirements, raw data are not available for public access, and data access should be discussed with the corresponding author.

## Supplementary Figure

Supplementary Figure.
