# Supplementary material for: Reconsidering screening thresholds in health assessments for obstructive sleep apnea using operational and safety incident data
Source: Sci Rep. 2024 May 13;14:10844. doi: 10.1038/s41598-024-61118-y (PMC11089039; doi:10.1038/s41598-024-61118-y)
Supplement: Supplementary file 1 — Supplementary Figure. [file 41598_2024_61118_MOESM1_ESM.docx]

# Supplementary material

Reconsidering screening thresholds in health assessments for Obstructive Sleep Apnea using operational and safety incident data

# Anjum Naweed^1*^, Bastien Lechat^2^, Janine Chapman^1^, Robert J. Adams^2,3^, Sally A. Ferguson^1^, Armand Casolin^4^ and Amy C. Reynolds^1,2^

^1^ Appleton Institute for Behavioural Science, Central Queensland University, Wayville, SA 5034, Australia

^2^ Flinders Health and Medical Research Institute (Sleep Health), Flinders University

^3^ Respiratory, Sleep and Ventilation Service, Southern Adelaide Local Health Network, SA Health

^4^ Transport for NSW, Macquarie Park, New South Wales

*anjum.naweed@cqu.edu.au

**Supplementary Figure 1.** Number of assessments per worker in the 2016-2018 health assessment window.
